# Time-series data from a hybrid finite element Large Eddy Simulation of flow over a backward-facing step

**DOI:** 10.1016/j.dib.2024.110330

**Published:** 2024-03-16

**Authors:** Angus C.W. Creech, Adrian Jackson

**Affiliations:** a Department of Mathematics and Statistics, Acadia University, 15 University Avenue Wolfville, Nova Scotia B4P 2R6, Canada; b The University of Edinburgh, Bayes Centre, 47 Potterrow, Edinburgh EH8 9BT, Scotland

**Keywords:** Computational fluid dynamics, Large Eddy Simulation, Backward-facing step, Finite elements, Discontinuous Galerkin

## Abstract

In previous work, a Large Eddy Simulation turbulence (LES) algorithm was developed for finite element-based computational fluid dynamics, using a hybrid continuous-discontinuous Galerkin scheme. The test case for this was a backward-facing step, which is a well-known example with published experimental results for validation. The results of this simulation were used to produce temporal statistics, such as averaged velocity and Reynolds stress profiles, and time-series data. In conjunction with previous published work by the authors, this data here will allow validation of other LES models and as well as detailed comparisons with the original model.

Specifications Table

**Table:** 

Subject	Hydrodynamics
Specific subject area	Technical data from Large Eddy Simulation of a backward-facing step
Data format	Raw
Type of data	CSV files, GMSH geometry and mesh files, Figures
Data collection	The results data has been calculated from a simulation of a backward-facing step with LES turbulence modelling [1], using an in-house finite element CFD code called CoastED, which itself is based upon Fluidity from Imperial College London. The geometry and mesh files were created with GMSH, an open-source mesh generation tool.
Data source location	Institution: EPCC, University of Edinburgh City: Edinburgh Country: Scotland/UK
Data accessibility	Repository name: Mendeley Data Data identification number: 10.17632/dnrckm26zc.1 Direct URL to data: https://data.mendeley.com/datasets/dnrckm26zc/1
Related research article	A.C.W. Creech and A. Jackson, Hybrid Large Eddy Simulation for low-order Discontinuous Galerkin methods using an explicit filter, Computer Physics Communications 260 (2021)

## 1 Value of the Data

• This data allows researchers to benchmark new turbulence models in computational fluid dynamics software with more precision than previously possible. In particular, it gives detailed time-series data from a simulation test case [1], which allows comparison with transient flow data, rather than just statistical data, such as time-average velocity plots and Reynolds stresses.
• Researchers who develop new turbulence models or new formulations of turbulence models in computational fluid dynamics.
• This data can be reused in three ways:
  i. The domain geometry information used by researchers to generate an exact reproduction of the simulation domain
  ii. The inlet velocity and Reynolds Stress profiles can be used to recreate inlet boundary conditions using the Synthetic Eddy Method
  iii. The statistical and time-series results data can be used for detailed comparison of both statistically-steady and transient flow information.

## 2 Background

This paper outlines a computational fluid dynamics dataset that provides LES simulation data of a reference benchmark case from a methodology paper published on a new LES model. In the parent paper we describe the new model and validate that model against other LES implementations using the backwards facing step testcase. Here we provide the output data from our simulation to allow others who are looking to compare their LES implementations on backwards facing step data to another implementation of the same functionality.

Detailed comparison and validation of LES models requires generation of significant amounts transient flow field information, which entails knowledge of alternative LES applications and the investment of time and computational resources in running comparator simulations. We provide time-series of velocity field profiles from our simulations as a reference dataset to enable others to re-use the data we have generated, enabling quick and easy comparisons to be undertaken. This complements the statistics commonly used for validation of such simulation models, such as time averaged velocity profiles and Reynold stresses.

## 3 Data Description

There are two sets of data: the geometry files and the data probe files.

The geometry files are used with the open-source mesh generation tool, GMSH. There is one for the domain geometry, and one for the final simulation mesh:

• backstep.geo
• adapted_mesh.msh

As for the data probe files, each contains the velocity and LES subgrid eddy viscosity, as described in EXPERIMENTAL DESIGN, MATERIALS AND METHODS. There are five files, one for a horizontal profile running streamwise in the centre of the domain, and four for vertical profiles which are also in the centre of the domain, perpendicular to horizontal profile and running from the bottom of the domain to the top. The files are named in line with the data they contain, as follows:

• horz-timeseries.csv
• vert-timeseries-*x* = 4H.csv
• vert-timeseries-*x* = 6H.csv
• vert-timeseries-*x* = 10H.csv
• vert-timeseries-*x* = 15H.csv

The ‘H’ stands for the height of the step, so ‘*x* = 4H’ means 4H downstream of that step. The specifics of each are described in the following sections. Each file is a comma separated file, with the first line in the file containing the column names for each data point. These are described in the sections below.

### 3.1 Geometry files

The geometry file contain files are compatible with the open-source mesh generation tool GMSH [2].

**Fig. 1:**
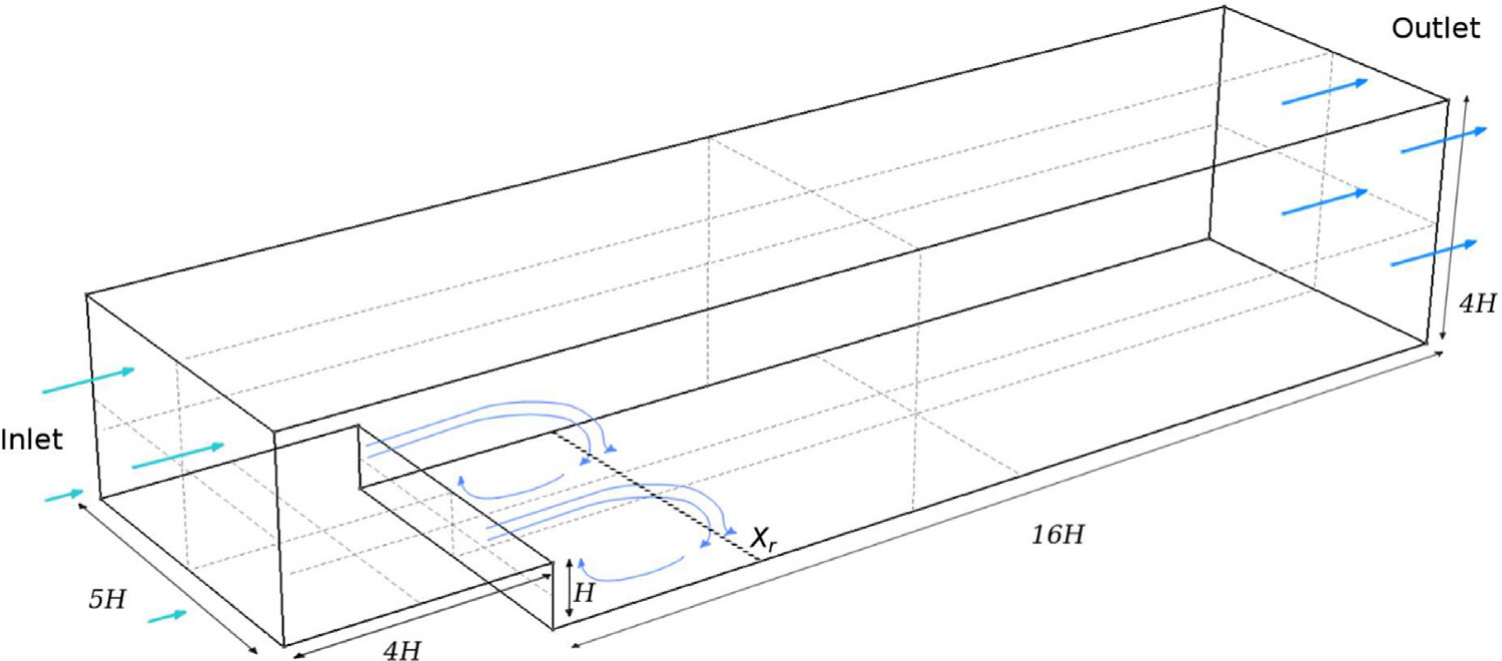
Backward-facing step model with dimensions, showing the reattachment point X_r_ beyond the step. Above this point the flow recirculates towards the step. Courtesy of Creech et al. [1].
(2) **adapted_mesh.msh** contained the final tetrahedral mesh which has been refined so that the criteria *y*^+^<1 holds at non-slip boundaries, and is in the GMSH mesh format. This can be used as a reference mesh to ensure resolutions are comparable with the original simulations. Alternatively, it may possible to import it into CFD software where appropriate tools are available, such as OpenFOAM's *gmshToFoam*. Please note the authors have not tested this file with OpenFOAM.

(1) **backstep.geo** contains the problem geometry in GMSH format. It is a text file that contains parameters to control the problem geometry, such as step height (*stepheight*), step length (*steplen*), domain length (*xsize*), width (*ysize*) and height (*height*). The vertical coordinate z equals 0 at the bottom of the deeper part of the channel downstream of the step. Running this with GMSH can produce the initial mesh. The geometry is shown in Fig. 1.

### 3.2 Horizontal profile (horz-timeseries.csv)

This data will allow the calculation of the reattachment (or recirculation) point, $X_{r}$. The horizontal, streamwise probe (or sample) points constitute a line downstream of the backward-facing step, in the centre of the domain and close to the bottom no-slip boundary.

The file has 7 columns of data:

(1) **t** : time in seconds.

(2) **x_H** : horizontal coordinate of probe point, with the backward-facing step as the origin, in units of H.

(3) **z_H** : vertical coordinate, also in units of H.
(4) **u_ms** : Cartesian x-component of velocity in units of ms^−1^.
(5) **v_ms** : Cartesian y-component of velocity in units of ms^−1^.
(6) **w_ms**: Cartesian z-component of velocity in units of ms^−1^.
(7) **nu_m2s** : the subgrid eddy viscosity υ in units of m^2^ *s*^−1^

There is one row per probe sample, and 13 points along the probe line with the data arranged so that for each timestep, there are 13 rows, one for each point, before the next timestep data appears, eg.:

t1, z1, …

t1, z2, …

…

t1, z13, …

t2, z1, …

For each probe point, there are 27,666 samples, starting from at a time of 36,009.5 s, and finishing at 155,342.0 s, giving a total sampling period of 119,332.5 s. This gives an average timestep of 4.3 s.

Vertical profile (vert-timeseries-*x*=*H.csv)

The vertical profiles are in the centre of the domain, placed at successive horizontal (x) distances downstream from the backward-facing step. Each file contains the data for one profile (ie. One line of probe points), as indicated by the filename. These distances are, in units of H: 4, 6, 10, 15.

Each file has 6 columns. These are:

(1) **t** : time in seconds.
(2) **z_H** : vertical coordinate in units of H.
(3) **u_ms** : Cartesian x-component of velocity in units of ms^−1^.
(4) **v_ms** : Cartesian y-component of velocity in units of ms^−1^.
(5) **w_ms**: Cartesian z-component of velocity in units of ms^−1^.
(6) **nu_m2s** : the subgrid eddy viscosity υ, in units of m^2^s^−1^

Each line consists of 17 points, with the specific location of those points described in the EXPERIMENTAL DESIGN, MATERIALS AND METHODS section following. As with the horizontal profile data, in each file there is one row per probe point per timestep, with 17 successive rows covering the probe line at each timestep. As with the horizontal profile probes, there are 27,666 samples per probe point, starting and ending at the same times, with the same timestep sizes.

**Fig. 2:**
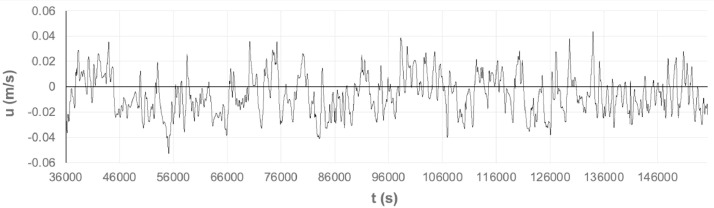
Time-series of u in vertical profile probe point at *x* = 6 H and *z* = 0.099 H.

**Fig. 3:**
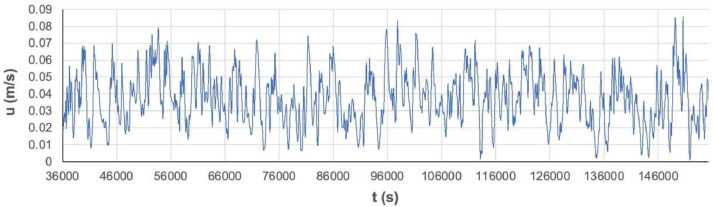
Time-series of u in vertical profile probe point at *x* = 10 H and *z* = 0.099 H.

Fig. 2, Fig. 3 show the time-series of u, for probe points at 6H and 10H downstream of the backward-facing step.

## 4 Experimental Design, Materials and Methods

**Fig. 4:**
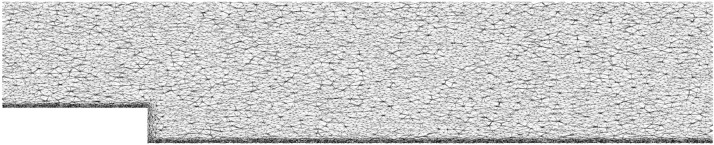
A streamwise vertical slice through the simulation domain, showing the final adapted mesh resolution.

The new LES formulation in Creech and Jackson [1] was developed for the open-source CFD package CoastED [3,4], a fork of Fluidity from Imperial College [5]; full details of the algorithm can be found in Section 2.3 of the original paper [1]. The turbulence model was tested against the well-known backward facing step, which consists of a rectilinear channel with a step of height Hat a distance 4Hfrom the inlet. The channel is 20H long, 5Hwide, 3H high at the inlet and 4H high at the outlet, as shown in Fig. 1. To match the experiments by Jovic and Driver [6] and simulations by Aider et al. [7], the Reynolds number was defined through the step height H, as $Re_{H}=5000_{\;}$. An initial mesh was generated by GMSH, which was then passed through the adaptive meshing routines in CoastED, to produce the final adapted mesh used for simulation. This consisted of an unstructured mesh of 913,060 tetrahedral cells, with the resolution increasing considerably near the no-slip bottom boundary: the first layer of elements was within the *y*^+^<1 wall-coordinate criteria and were broadly isotropic. As the distance from the no-slip boundary increased, elements became mildly anisotropic, and the horizontal-vertical aspect ratio increased to 4:1. A vertical streamwise slice through the mesh can be seen in Fig. 4.

A P1DG-P2 velocity-pressure finite element representation was used for the velocity and pressure fields. The velocity elements were Compact Discontinuous Galerkin, similar to Peraire and Persson [8], with a twice-Picard iterated approximation to the Crank-Nicolson scheme for time-stepping [9]. Tests on adaptive time-stepping gave a maximum allowable Courant-Fredrichs-Levy condition of CFL<1.6. The bottom of the channel has a no-slip boundary condition, and the sides and top are frictionless (free-slip). As with Patil [10], the inlet had a synthetic eddy method boundary condition [11] which placed turbulent fluctuations on top of a logarithmic velocity profile. The outlet is an open boundary with a Von Neumann condition, ie. ∇u=0.

For the synthetic eddy method, the turbulent inflow conditions used a logarithmic profile for the mean velocity profile based upon flow measurements by Nezu and Nakagawa [12]:

$$\bar{u}\left(z_{s}\right)=\left(\frac{u_{\tau }}{K}\right)\;\text{log}\left(\frac{z_{ref}+z_{R}}{z_{R}}\right)+8.5$$

where $u_{\tau }$ is the frictional velocity, K≈0.41 is the von Karman constant, $z_{s}$ is the height above the step, $z_{ref}\;$is the height at which we reference the free-stream flow speed $u_{O}$, and $z_{R}$ is the roughness height. The mean cross-stream and vertical components were set so that $\bar{v}=\bar{w}=0$.

We can define $u_{\tau }$ as:

$$u_{\tau }=\left(\frac{u_{0}}{K}\right)\;\text{log}{\left(\frac{z_{ref}+z_{R}}{z_{R}}+8.5\right)}^{-1}$$

For our simulations, the roughness height was chosen to be very small compared to the step, with $z_{R}=0.0001$. The reference height for $u_{O}$ was chosen to be $z_{ref}=H/4$. The diagonal components of the Reynolds Stress $Re_{ii}$ need to be specified for synthetic eddy method; these were based on Stacey et al. [13], which following on from [12] gives:

$$R_{uu}=\bar{u^{\prime }u^{\prime }}=5.28\;{u_{\tau }}^{2}\;\text{exp}\left(-\frac{2z}{H_{in}}\right)$$

$$R_{vv}=\bar{v^{\prime }v^{\prime }}=2.66\;{u_{\tau }}^{2}\;\text{exp}\left(-\frac{2z}{H_{in}}\right)$$

$$R_{ww}=\bar{w^{\prime }w^{\prime }}=1.61\;{u_{\tau }}^{2}\;\text{exp}\left(-\frac{2z}{H_{in}}\right)$$

where $H_{in}\;$*i*s the height of the channel at the inlet. The turbulence length scale components, $L_{u}$, $L_{v}\;$and $L_{w}\;$were again derived from Nezu and Nakagawa [12], thus or the bottom half of the inlet the streamwise eddy length scale is:

$$L_{u}=\sqrt{zH_{in}}$$

With $L_{u}=\frac{1}{2}H_{\text{in}}$ for the top half; the cross-stream and vertical components are defined as:

$$\begin{array}{ccc} L_{v} & = & \frac{1}{2}L_{u}\\ L_{w} & = & \frac{1}{4}L_{u} \end{array}$$

Results for the spin-up phase of the simulation were discarded, and the simulation run for sufficient time to ensure that calculations for time-averaged velocity and Reynolds stress values were statistically stationary. Two sets of sampling points were defined in the simulation, which would output velocity and the subgrid eddy viscosity at each timestep. The first set consisted of a horizontal line of sample points placed streamwise in the centre of the domain, downstream of the backward-facing step, described in Table 1.

**Table 1:** Position of the horizontal line sample points with respect to H. Vertical position is with respect to the bottom boundary of the simulation domain and horizontal position from the inflow boundary.

Data point	Vertical Position (H)	Horizontal Position (H)
1	0.25	1
2	0.25	2
3	0.25	3
4	0.25	4
5	0.25	5
6	0.25	6
7	0.25	7
8	0.25	8
9	0.25	9
10	0.25	10
11	0.25	12
12	0.25	14
13	0.25	16

**Fig. 5:**
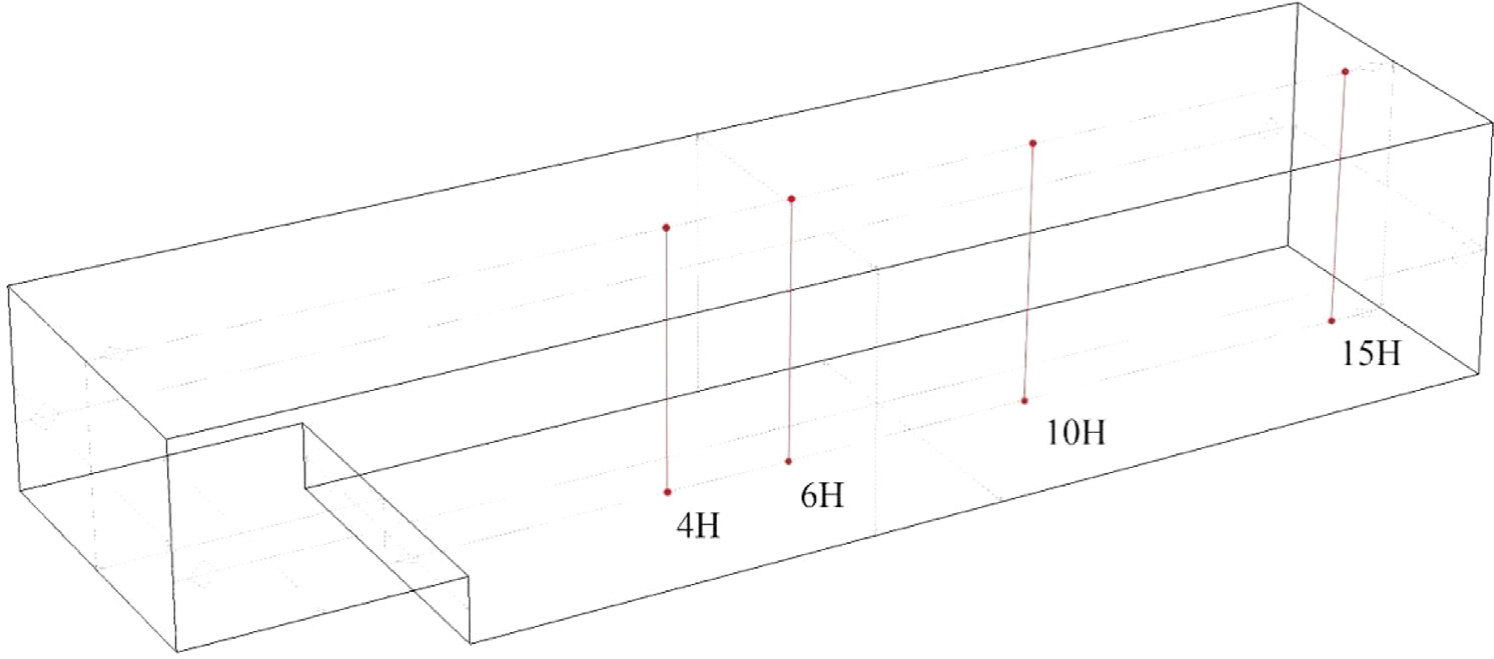
Backward-facing step domain showing vertical sample point profiles at *x* = 4H, 6H, 10H and 15H.

The second set consisted of a series of sample points arranged in vertical lines, also in the centre of the domain, which were placed at distances of 4H, 6H, 10H and 15H downstream of the backward-facing step, and reached from the bottom of the domain to the top (see Fig. 5 and Table 2).

**Table 2:** Vertical position for each point in a vertical sample point with respect to H from the bottle of the domain.

Data Point (Per Profile)	Vertical Position (H)
1	0.025510204
2	0.050000000
3	0.098979592
4	0.197959184
5	0.395918367
6	0.593877551
7	0.791836735
8	0.989795918
9	1.187755102
10	1.385714286
11	1.583673469
12	1.781632653
13	1.979591837
14	2.177551020
15	2.474489796
16	2.969387755
17	3.959183673

To ascertain the recirculation point$\;X_{r}$, time-averaged velocity values were taken from the horizontal sample points shown in Table 1 to find the point of zero velocity. Comparative values of $X_{r}$ can be seen in Table 3.

**Table 3:** Comparison of selected values of X_r_. Jovic and Driver [6] represents the experimental data; Creech and Jackson [1] represents the model that produced the data in this paper.

Source	$X_{r}$ (H)	Rel. error (%)
Jovic and Driver [6]	6.0	–
Le et al. (1993) [14]	6.0	0
Creech and Jackson [1]	6.065	1.072
Aider et al. – WN [7]	5.8	3.448
Le et al. (1997) [15]	6.28	4.459
Aider et al – PS [7]	5.29	11.833
Panjwani et al. [16]	7.2	16.666
Dubief and Delcayre [17]	7.2	16.666

Simulations in CoastED typically used 96 cores and took approximately 10 h to produce the data [18].

## Limitations

None.

## Ethics Statement

The authors have read and followed the ethical requirements for publication in this journal. Our data presented here contains no data from experiments with human or animal subjects, nor has it been collected from social media.

## CRediT authorship contribution statement

**Angus C.W. Creech:** Conceptualization, Data curation, Methodology, Visualization, Writing – review & editing. **Adrian Jackson:** Resources, Validation, Writing – review & editing.

## Data Availability

Velocity time-series data from a hybrid finite element Large Eddy Simulation of flow over a backward-facing step (Original data) (Mendeley Data)
